# Neurophysiological and behavioural effects of conventional and high definition tDCS

**DOI:** 10.1038/s41598-021-87371-z

**Published:** 2021-04-07

**Authors:** Fabio Masina, Giorgio Arcara, Eleonora Galletti, Isabella Cinque, Luciano Gamberini, Daniela Mapelli

**Affiliations:** 1 IRCCS San Camillo Hospital, Venice, Italy; 2 Human Inspired Technologies Research Center, University of Padova, Padua, Italy; 3 Department of General Psychology, University of Padova, Padua, Italy

**Keywords:** Neuroscience, Psychology

## Abstract

High-definition transcranial direct current stimulation (HD-tDCS) seems to overcome a drawback of traditional bipolar tDCS: the wide-spread diffusion of the electric field. Nevertheless, most of the differences that characterise the two techniques are based on mathematical simulations and not on real, behavioural and neurophysiological, data. The study aims to compare a widespread tDCS montage (i.e., a Conventional bipolar montage with extracephalic return electrode) and HD-tDCS, investigating differences both at a behavioural level, in terms of dexterity performance, and a neurophysiological level, as modifications of alpha and beta power as measured with EEG. Thirty participants took part in three sessions, one for each montage: Conventional tDCS, HD-tDCS, and sham. In all the conditions, the anode was placed over C4, while the cathode/s placed according to the montage. At baseline, during, and after each stimulation condition, dexterity was assessed with a Finger Tapping Task. In addition, resting-state EEG was recorded at baseline and after the stimulation. Power spectrum density was calculated, selecting two frequency bands: alpha (8–12 Hz) and beta (18–22 Hz). Linear mixed effect models (LMMs) were used to analyse the modulation induced by tDCS. To evaluate differences among the montages and consider state-dependency phenomenon, the post-stimulation measurements were covariate-adjusted for baseline levels. We observed that HD-tDCS induced an alpha power reduction in participants with lower alpha at baseline. Conversely, Conventional tDCS induced a beta power reduction in participants with higher beta at baseline. Furthermore, data showed a trend towards a behavioural effect of HD-tDCS in participants with lower beta at baseline showing faster response times. Conventional and HD-tDCS distinctively modulated cortical activity. The study highlights the importance of considering state-dependency to determine the effects of tDCS on individuals.

## Introduction

Transcranial direct current stimulation (tDCS) is a non-invasive brain stimulation (NIBS) technique that uses a constant weak electric current (generally 1–2 mA) to modulate specific brain areas over which it is applied^1–3^. The current passes through the scalp, skull, and cerebrospinal fluid down to the cortex, bringing membrane potential of neurons closer to or farther from the action potential threshold^4^. Typically, in bipolar tDCS the current is delivered through two electrodes, an anode and a cathode, normally sized 25–35 cm^2^^1^. One electrode is placed over the area of interest, while the other one is placed over another site, either cephalic or extracephalic^5^.

Despite the growing popularity of tDCS, especially as a promising tool for the treatment of clinical conditions, several criticisms have undermined the reliability of this technique: the inconsistency of tDCS effects and the difficulty to replicate previous findings^6^. The reasons behind the crisis of tDCS are related to several aspects, mostly methodological, which likely had a substantial impact in the replication of the results^7, 8^.

A further critical aspect is related to biophysical properties and concerns the spatial distribution of the electric field of bipolar tDCS montages. As a matter of fact, bipolar tDCS produces a wide-spread electric field with low spatial specificity and peaks of intensity falling outside the active electrode^9, 10^. Since the stimulation reaches brain areas that are structurally and functionally different, the electric field distribution may be another issue explaining such variability and inconsistency of tDCS-induced effects at both behavioural and neurophysiological levels.

Recently, new tDCS montages and devices have been introduced to overcome the limitation of bipolar tDCS (henceforth “Conventional”). High-definition tDCS (HD-tDCS) montages use smaller electrodes than Conventional tDCS allowing the electric current to be delivered with increased density and focality. The most used HD-tDCS montage is the 4 × 1 ring configuration, which consists of one active electrode placed on the area of interest and four return electrodes on the surroundings. In this way, the delivered electric current is constrained and localised within the return electrodes^9^. Finite element method (FEM) models predict a different strength and distribution of electric field induced by Conventional and HD-tDCS^10–12^. In particular, specific electrode placements of HD-tDCS contribute to reduce the uncontrolled diffusion of tDCS-induced electric fields, thereby improving the spatial precision with which the electrical current can target specific cortical regions^13^. Findings coming from physiological studies have also presented a higher precision of HD-tDCS in modulating neurophysiological components as compared to Conventional tDCS^9, 14^.

Although different electric field distributions have been found in computational studies concerning Conventional and HD-tDCS, no clear conclusion can be drawn on whether HD-tDCS can really produce a more focal and strong modulation than Conventional tDCS, both at behavioural and neurophysiological levels^15, 16^. Indeed, most of the differences between the two techniques are based on mathematical simulations, and not on empirical effects of the stimulation. Only few studies have directly investigated the effects of HD-tDCS since its introduction in the NIBS field. However, these studies typically consider either the behavioural and EEG effects of HD-tDCS, without a direct comparison of this montage with a Conventional tDCS montage (for example^17, 18^) or compare in the same study both the montages, without providing EEG measures (for example^19, 20^).

To fill this gap and to further elucidate the differential effects of Conventional and HD-tDCS, the main goal of this research is to compare these two montages, investigating in the same study, for the first time in healthy participants, both behavioural and neurophysiological outcomes. Importantly, to evaluate tDCS effects, this study capitalises on some methodological advances as compared to common standards. The large majority of studies using tDCS investigate effects by comparing baseline and post-stimulation measurements^21, 22^, or by using change scores, that is analysing effects obtained by subtracting baseline from post-stimulation measurements^23, 24^. These widespread approaches assume several hidden statistical assumptions (often not satisfied) and ignore some well-known potential distortions. One of these is the phenomenon of regression to the mean^25, 26^, according to which extreme values (very low or very high) tend to be closer to the mean in a repeated measure for pure statistical reasons. Moreover, baseline levels can constraint potential outcome of treatment also for other reasons. For example, if the behavioural scores are already at ceiling at baseline, no further improvement is possible, and this cannot be explicitly taken into account using mean scores or change scores in the statistical analysis (as it happens with *t*-test or ANOVA).

To overcome these limitations and following gold standard for randomised control treatment design in biostatistics^25^, in the present study post-stimulation measurements were adjusted for baseline levels, considering these latter as covariates in statistical models. By using this approach, the research question changes from “Is HD-tDCS better than Conventional tDCS?” to “Are there specific conditions under which HD-tDCS is better than Conventional tDCS?”. This is a methodological shift towards addressing more proper questions, as the investigation of tDCS effects cannot ignore the potential of observing actual modifications which highly depend on baseline values. Remarkably, applying this rationale and method to neural data (e.g., EEG data) allows to properly control for the initial activation state of brain before the stimulation, hence taking into account the phenomenon of state-dependency^2, 27^. Although several articles advocate the importance of state-dependency in explaining the variability of observed effects or the failure of observing significant effects^2, 28^, yet few studies actually take into account initial state as a variable in the analysis, so explicitly investigating the actual influence of state-dependency effects.

In the present study, tDCS was used in combination with a motor task given the large body of evidence suggesting the technique would induce the strongest effects within the motor domain (for a review and meta-analysis, see^29^). In particular, tDCS was delivered with the aim to modulate participant’s dexterity, which was assessed with a computerised version of the Finger Tapping Task, administered before, during, and after the stimulation stage. Furthermore, the Purdue Pegboard Test^30^ was used to assess dexterity in a non-computer based fashion, also controlling if tDCS effects could be generalized to a more ecological measure than the Finger Tapping Task. We expected that for the HD-tDCS condition we would observe a greater improvement in participants’ dexterity relative to Conventional tDCS and sham. Furthermore, we expected greater dexterity improvement in Conventional tDCS relative to sham.

At a neurophysiological level, resting-state EEG was recorded before and after the stimulation stage. Power spectrum density was calculated selecting two EEG bands, respectively alpha (8–12 Hz) and beta band (18–22 Hz). The rationale of selecting alpha and beta bands is based on the functional role of these two bands. While the alpha frequency band has been linked with general inhibitory mechanisms^31^, thus linking the decrease of alpha power to motor facilitation, beta power has been related to sensory-motor functions^32^.

A recent review^33^ shows that anodal tDCS reduces alpha power while increasing beta power. For this reason, we expected that anodal HD-tDCS would produce a greater modulation of the neurophysiological signal, leading to a significantly greater decrease of alpha and increase of beta power as compared to the Conventional and sham conditions. Moreover, we expected that Conventional tDCS would cause a greater modulation relative to sham.

## Results

### The Finger Tapping Task

The mean response times (RTs) and accuracy for each stimulation condition are summarised in Table 1.

**Table 1:** Mean and standard deviation (*SD*) of performance measures on the Finger Tapping Task and the Purdue Pegboard Test for Conventional tDCS, HD-tDCS, and sham.

Measures	Conventional tDCS			HD-tDCS			Sham		
	Baseline	Stimulation	Post-stimulation	Baseline	Stimulation	Post-stimulation	Baseline	Stimulation	Post-stimulation
FTT—RTs (ms)	1956 (810)	1745 (651)	1582 (615)	1996 (798)	1792 (675)	1582 (572)	2024 (904)	1780 (717)	1663 (619)
FTT—accuracy (%)	87.9 (7.7)	86.9 (5.9)	88 (8.6)	87.6 (9.1)	85.8 (6.4)	88.9 (6)	87.8 (8.1)	87.3 (6.3)	85.9 (8.7)
PPT—left hand	–	–	14.7 (1.6)	–	–	14.3 (1.6)	–	–	14.4 (1.5)

*RTs* response times, *FTT* Finger Tapping Task, *PPT* Purdue Pegboard Test.

**Figure 1:**
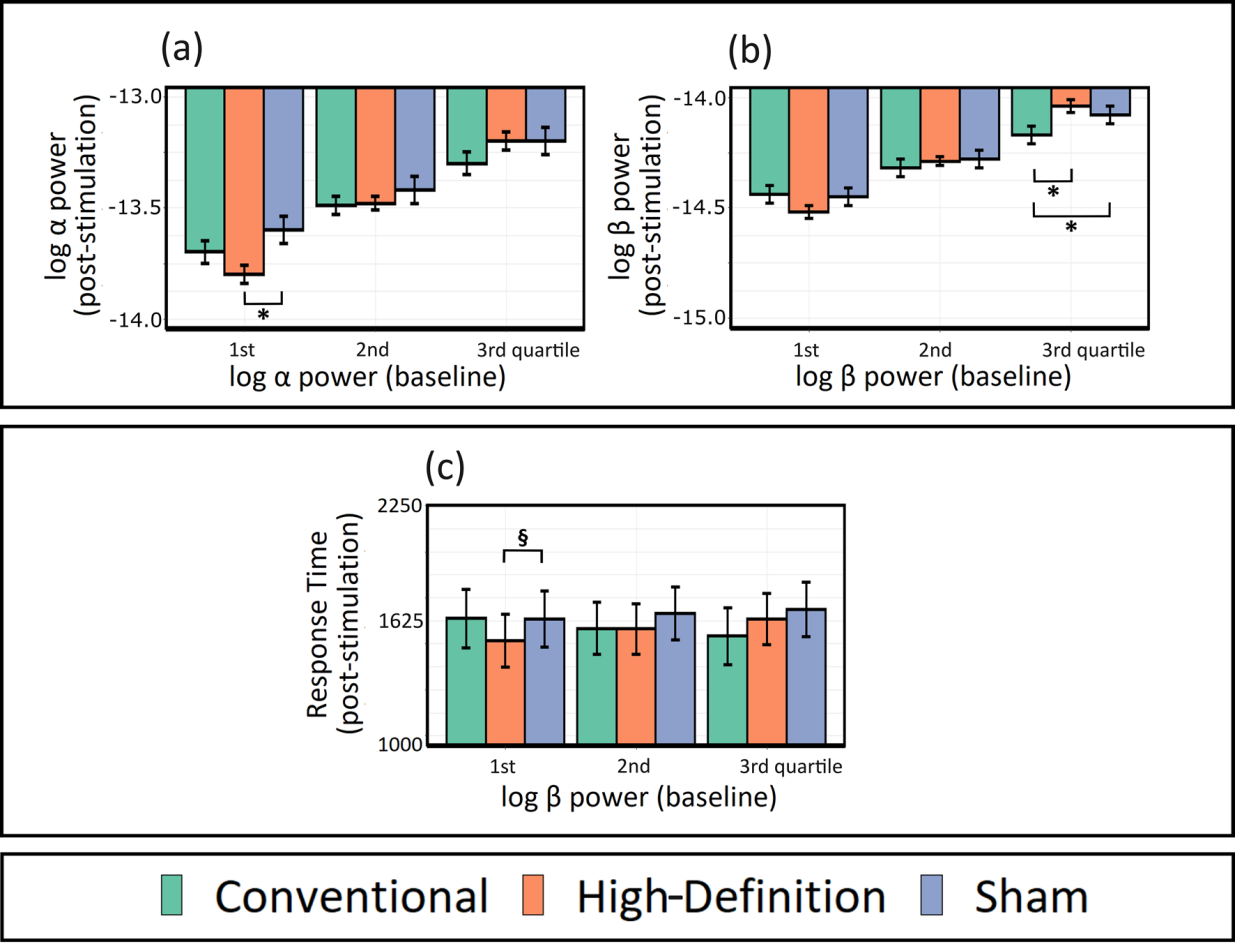
Neurophysiological and behavioural results. (**a**) Modulation of alpha power in the post-stimulation stage across stimulation conditions with alpha power at the baseline as covariate. In the x-axis, the 1st, 2nd, and 3rd quartile of the covariate, respectively − 14, − 13.5, − 13.2 power units, while in the y-axis, alpha power at the post-stimulation stage. At low levels of alpha power at the baseline (i.e., the 1st quartile), results show a significant reduction of alpha power in the post-stimulation stage only for HD-tDCS (red bar) relative to sham (blue bar). (**b**) Modulation of beta power in the post-stimulation stage across stimulation conditions with beta power at the baseline as covariate. The x-axis shows the 1st, 2nd, and 3rd quartile of the covariate, respectively − 14.6, − 14.3, − 13.9 power units, while the y-axis represents beta power at the post-stimulation stage. At high levels of beta power at the baseline (i.e., the 3rd quartile), a significant reduction of beta power in the post-stimulation stage was found only for Conventional tDCS (green bar) relative to both HD-tDCS and sham. (**c**) Response times (RTs) modulation across stimulation conditions with beta power at the baseline stage as covariate. In the x-axis, the 1st, 2nd, and 3rd quartile of the covariate, namely − 14.6, − 14.3, − 14 power units, while in the y-axis, RTs at the Finger Tapping Task performed during the post-stimulation stage. At low levels of beta power in the baseline stage (i.e., the 1st quartile), results show a reduction of RTs in HD-tDCS relative to sham. *p < 0.05; ^§^*p* = 0.068.

With regard to RTs, in the model with beta power entered as covariate, a main effect of *stimulation condition* was found [*F*(2,29.9) = 3.51, *p* = 0.043]. Post-hoc contrasts did not show significant differences between the conditions (lowest *p* = 0.113). In this model, a significant *stimulation condition *× *beta power* at *baseline stage* interaction was found [*F*(2,29.9) = 3.58, *p* = 0.04], (Fig. 1). Contrasts were performed at the level of the 1st, 2nd, and 3rd quartile of beta power at the baseline stage, respectively − 14.6, − 14.3, − 14 power units. Post-hoc tests showed a tendency towards significance: for lower beta at baseline (i.e., − 14.6 power units), HD-tDCS reduced RTs compared to sham (1514 ms vs. 1634 ms, *p* = 0.068). No effect was identified in the models that considered as covariate alpha power, as well as all the models on accuracy (see supplementary material). Results from the additional models (not covariate-adjusted) including the factors *task stage* (baseline, stimulation, and post-stimulation stage) and *stimulation condition* (Convention tDCS, HD-tDCS, and sham) are discussed in supplementary material.

### The Purdue Pegboard Test

The mean performance at the Purdue Pegboard Test for each stimulation condition is shown in Table 1. The models considered in the analysis did not show any significant effects (see supplementary material).

### EEG analysis

The model investigating the impact of tDCS on alpha power showed several significant effects. *F*-tests showed a main effect of *stimulation condition* [*F*(2,52.9) = 9.69, *p* < 0.001], despite post-hoc contrasts did not show any significant differences among the conditions (lowest *p* = 0.203). In addition, a *stimulation condition *× *alpha power baseline stage* interaction was found [*F*(2,52.9) = 9.87, *p* < 0.001], (Fig. 1). Contrasts were conducted at the level of the 1st, 2nd, and 3rd quartile of alpha power at the baseline stage, respectively − 14, − 13.5, − 13.2 power units. Post-hoc tests showed that for lower power values of this covariate (i.e., − 14 power units), HD-tDCS reduced alpha power compared to sham (− 13.8 vs. − 13.6, *p* = 0.013).

In the second model, in which beta power was considered, analysis showed a main effect of *stimulation condition* [*F*(2,80.5) = 15.64, *p* < 0.001]. Post-hoc comparisons did not reveal a difference between the conditions (lowest *p* = 0.408). A significant interaction between *stimulation condition *× *beta power baseline stage* was found [*F*(2,80.7) = 15.45, *p* < 0.001], (Fig. 1). By conducting contrasts at the 1st, 2nd, and 3rd quartile of the covariate, respectively − 14.6, − 14.3, − 13.9 power units, a reduction of beta power was found in Conventional tDCS compared to HD-tDCS (− 14.2 vs. − 14, *p* = 0.002), and in Conventional tDCS compared to sham (− 14.2 vs. − 14.1, *p* = 0.045), for higher beta power values at the baseline stage (i.e., − 13.9 power units).

## Discussion

The present study aims to provide new evidence characterising functional differences between Conventional (i.e., bipolar tDCS with extracephalic return electrode) and HD-tDCS. To properly evaluate effects of both the montages, we adopted a state-of-the-art approach for the statistical analysis that overcomes some common suboptimal statistical methods and focalises on properly identifying the conditions associated with specific treatment outcome^25^. This approach consisted in adjusting the post-stimulation measurements for baseline levels, considering these latter as covariates in the analysis. Of importance, this method allowed to properly account for state-dependency^2, 34, 35^, a phenomenon often advocated to explain variability and inconsistency of tDCS-induced effects^36^, but generally neglected in statistical analysis.

Results from the present study outline distinctive modulation of Conventional and HD-tDCS. This evidence, observed empirically, is possibly related to the different strength and distribution of the electric field induced by Conventional and HD-tDCS, as predicted by FEM computational models^10–12^. Firstly, we found that following HD-tDCS, a reduction of EEG alpha power during resting state could be observed. This result is consistent with previous studies showing an inhibitory effect of anodal tDCS on alpha power^33^. The functional role of alpha is commonly related to cortical deactivation and inhibition^31, 37, 38^ and animal models provide evidence that alpha-band oscillations have an inhibitory influence on the generation of spikes^39^. Consequently, if an increase of alpha power reflects inhibition, a reduction in power should reflect release from inhibition, supporting evidence that anodal HD-tDCS on M1 induces cortical excitability^14^.

In line with our expectations, the state of neural activation before applying HD-tDCS played a role in the modulation of alpha. Specifically, a reduction of alpha power was observed only in participants that had lower EEG alpha before HD-tDCS. A possible explanation of this result may rely on the cognitive role linked to alpha band, namely its involvement in attention^31^. This viewpoint would suggest that only participants with a proper attentional asset (i.e., lower alpha and, consequently, higher level of attention before the stimulation) benefited from a release of inhibition following the administration of HD-tDCS. Remarkably, this result contributes to identify which physiological markers can predict tDCS effects, especially the effects of HD-tDCS that is relatively recent among NIBS techniques.

Contrary to our expectations, we found that Conventional tDCS induced a reduction of beta power. Specifically, in our study the reduction of beta occurred for participants who already showed a higher level of beta power before the administration of Conventional tDCS. The inhibition of beta induced by anodal tDCS is not unusual in the literature^40, 41^, despite previous evidence showing an opposite pattern, namely an increase of beta power^33^.

The inconsistency of these findings may be accounted for as a consequence of the uncontrolled spread of the electric field in Conventional tDCS^9^. As shown, in tDCS the current generally concentrates at the edge of the electrode^42^. Thus, the larger is the electrode size the lower is the probability to keep the edge of the electrode over the target area. With the unlikelihood of being able to control the electric current diffusion, a possible concern arises in the fact that tCDS may affect more than just the target region. Consequently, Conventional tDCS, which typically uses large electrodes sized 25–35 cm^2^, may lead to undesired or mixed effects due to the stimulation of nearby areas connected to the target area. Further studies are necessary to confirm this hypothesis, possibly by comparing functional outcomes of Conventional and HD-tDCS in the same study.

With respect to behavioural outcomes, HD-tDCS produced some preliminary evidence (i.e., a trend towards significance) of a potential motor improvement in participants who had lower beta power at the baseline stage. This finding would suggest that HD-tDCS can induce enhancement of unimanual dexterity, as shown by a previous study^43^. Functionally, beta power has been hypothesised to be linked to sensory-motor functions^32^. Generally, a reduction of beta power is seen in planning and execution of motor action, followed by an increase of power after the end of the movement^44, 45^. However, the dynamic fluctuations of beta power before, during, and after a voluntary movement must reflect several underlying processes. While the desynchronisation of beta (i.e., power decrease) would be related to the asynchronous activation of the motor cortex during a movement, the synchronization (i.e., power increase) seems to reflect several mechanisms including motor control^46^ and the maintenance of tonic activity at the cost of voluntary movements^47^. Interestingly, this latter hypothesis is coherent with a recent study showing that the increase of beta activity would induce a slowdown of movements^48^. Thus, the improvement in dexterity granted by HD-tDCS might prove to be higher if the brain state has an adequate level of disposition to the movement onset (i.e., lower beta power). Nevertheless, given the small effect found, further research is necessary to confirm the possibility to enhance dexterity by HD-tDCS, possibly adopting a different task or investigating clinical population, in which the risk of a ceiling effect is reduced and the potential to improve motor performance is higher^49^.

Of importance, from a statistical point of view, results of this study underline the importance of adjusting the post-stimulation measurements for baseline levels, including these latter as covariates in statistical models. This approach, in line with recent suggestions for randomised control treatment designs in biostatistics^25^, should be considered as a good practice in future NIBS studies, especially with the aim to investigate the effect of stimulation not only per se but also in relation to the initial state of brain, and to the actual potential to be influenced by tDCS.

In relation to the present study, some limitations should be mentioned. The first limitation regards the interpretation of our findings. Although the uncontrolled diffusion of the electric field may modulate non-targeted areas, leading to unwelcomed effects, it should also be recognized that widespread changes in brain physiology can also be given by the state of cortical activation at the time of stimulation^50^, regardless of the spreading of the electric field. In fact, tDCS can selectively modulate cortical regions far from the target area, involving task-related brain networks active while delivering stimulation^51–54^. Thus, also HD-tDCS, despite having a more focal electric field compared to Conventional tDCS, might induce physiological changes far from the target region, activating widespread networks functionally connected to the target area.

The second limitation relates to the absence of a comparison between HD-tDCS and a Conventional tDCS montage with a return electrode over a cephalic site. The position of the return electrode is critical because the distance between anode and cathode can be crucial in modulating the primary motor cortex^55^. This comparison would have allowed to extend the comprehension of brain physiology underlying tDCS-induced plasticity, considering in the same study two Conventional bipolar montages, both cephalic and extracephalic, and HD-tDCS. However, we did not adopt Conventional tDCS with cephalic return electrode to exclude that the effects of stimulation were due to a possible inhibitory effect of cathode placed on a not-inert brain site.

In summary, the present study is the first multimethod and multimodal study that compares, in healthy participants, Conventional and HD-tDCS, considering their effects both from a behavioural and neurophysiological perspective (i.e., by EEG). HD-tDCS represents a recent advance in NIBS since it would overcome the low precision of Conventional tDCS. However, few studies have compared whether the increased focality of HD-tDCS could determine different modulation. Interestingly, our findings support this claim and show how HD-tDCS can induce more predictable outcomes than Conventional tDCS. A further relevant achievement of this study regards the statistical approach used to consider tDCS-induced plasticity in a more sophisticated and proper way. This study properly controls for the initial activation state of brain before the stimulation, hence addressing the phenomenon of state-dependency. Significantly, the present study also highlights the importance of considering the initial state of brain activity before tDCS application, as it can be crucial to influence the effects of tDCS.

## Methods and materials

### Participants

Thirty participants were recruited from the University of Padua. They were matched for gender (15 males and 15 females) and their age range was between 19–30 years old (mean age = 23.4, standard deviation (*SD*) = 1.9; mean education = 16.2, *SD* = 1.3). All participants were right-handed, as indicated by the Edinburgh Handedness Inventory^56^ (mean laterality score: 80.5, *SD*: 16.8), and reported normal or corrected-to-normal visual acuity.

Participants with a history of neurological or psychiatric diseases were excluded from the study. They were all checked for tDCS exclusion criteria^57^. All safety procedures were in line with tDCS guidelines^57^. Before the experiment, participants gave their written informed consent. The study was approved by the ethics committee of the Human Inspired Technology (HIT) Research Centre in Padua (nr. 2019_39) and was compliant with the ethical principles of the 1964 Declaration of Helsinki.

### Transcranial direct current stimulation (tDCS)

The tDCS and EEG recordings were carried out through a multi-focal tDCS-EEG device (StarStim, Barcelona) with 20 channels. The system was remotely controlled via the Neuroelectrics Instrument Controller (NIC; v2.0.11.4). Participants were involved in three experimental sessions, during which a different tDCS montage was applied, namely Conventional tDCS or HD-tDCS.

**Figure 2:**
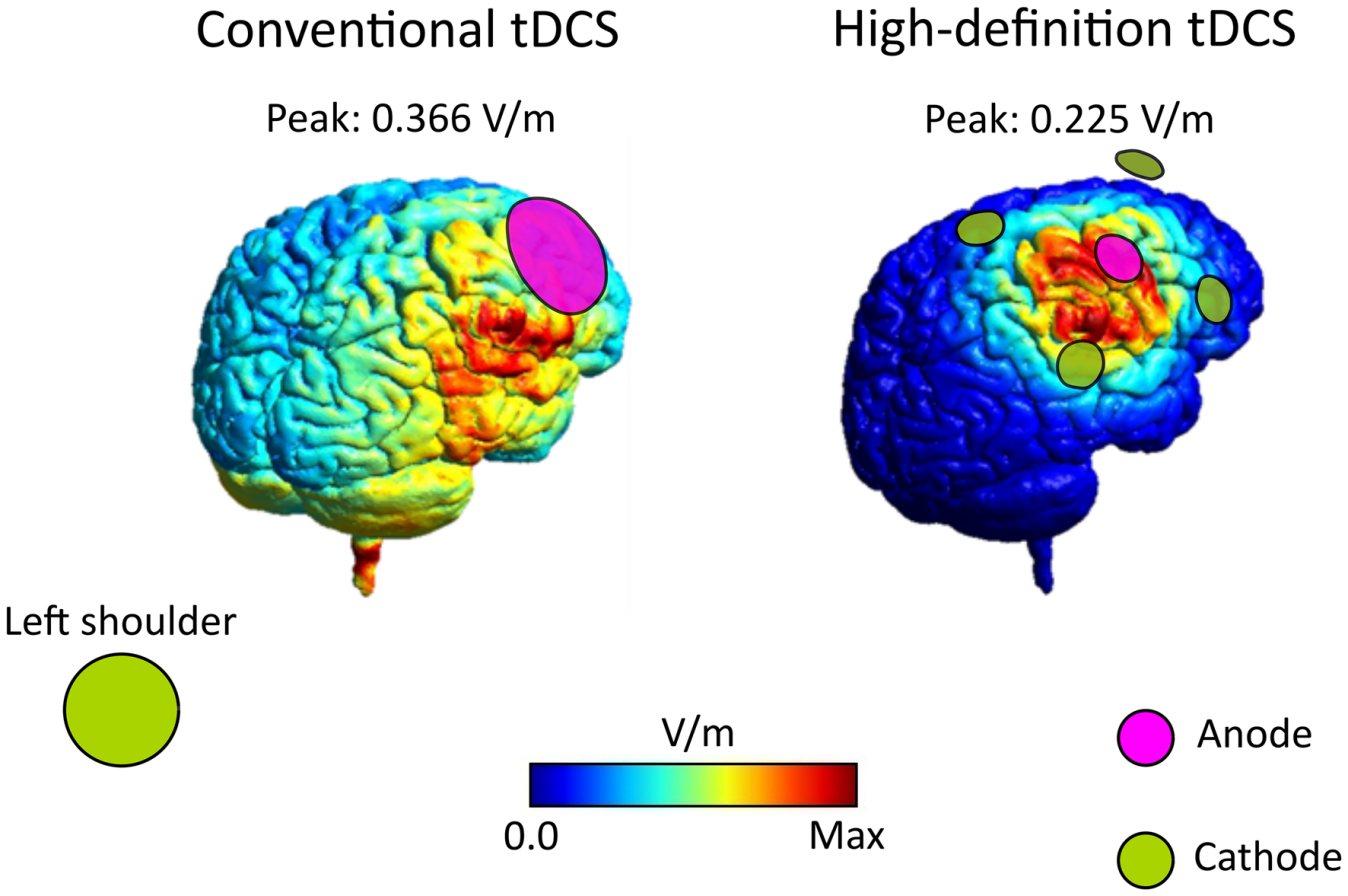
Conventional vs. HD-tDCS simulations. Finite element method (FEM) simulations showing the electric fields of Conventional and HD-tDCS montages, both estimated by SimNIBS^65^. In the image on the left, the electric field of Conventional tDCS (anode: C4; cathode: left shoulder). On the right, the electric field of HD-tDCS (anode: C4; cathodes: FC2, FC6, CP2, CP6). As shown, the electric field elicited by HD-tDCS is much more focal than Conventional tDCS where the current is more spread.

In Conventional tDCS, two circular saline-soaked surface sponge electrodes (surface = 25 cm^2^; current density: 0.06 mA/cm^2^) were used. The anode (active electrode) was placed on C4 (International 10–20 EEG System), while the cathode (return electrode) was placed over the contralateral (left) shoulder of participants. The shoulder is an extracephalic electrode position commonly used in tDCS studies (for example^58–61^) to avoid the confounding effects of placing the return electrode on a cephalic site^62, 63^. In HD-tDCS, a 4 × 1-ring configuration with sintered Ag/AgCl electrodes (surface = 3.14 cm^2^; current density: 0.48 mA/cm^2^) was adopted. The anode was placed over C4 and the four cathodes on FC2, FC6, CP2, CP6. In this electrode configuration, the electric current is assumed to be less spread than Conventional tDCS^9, 14, 64^, as shown in electric field models (Fig. 2, see also Figure S4 in supplementary material) simulated with SimNIBS^65^.

Along with the real tDCS, a sham condition was included where the montages were counterbalanced. Hence, 50% of participants received sham with the Conventional tDCS montage and the other 50% received sham with the HD-tDCS montage.

At the end of each session, participants completed a questionnaire of tDCS-related sensations^66^. Remarkably, participants were not able to distinguish between real tDCS and sham [session 1: Wald χ^2^(2) = 0.37, *p* = 0.83; session 2: Wald χ^2^(2) = 1.25, *p* = 0.53; session 3: Wald χ^2^(2) = 0.1.88, *p* = 0.392].

### EEG recording

**Figure 3:**
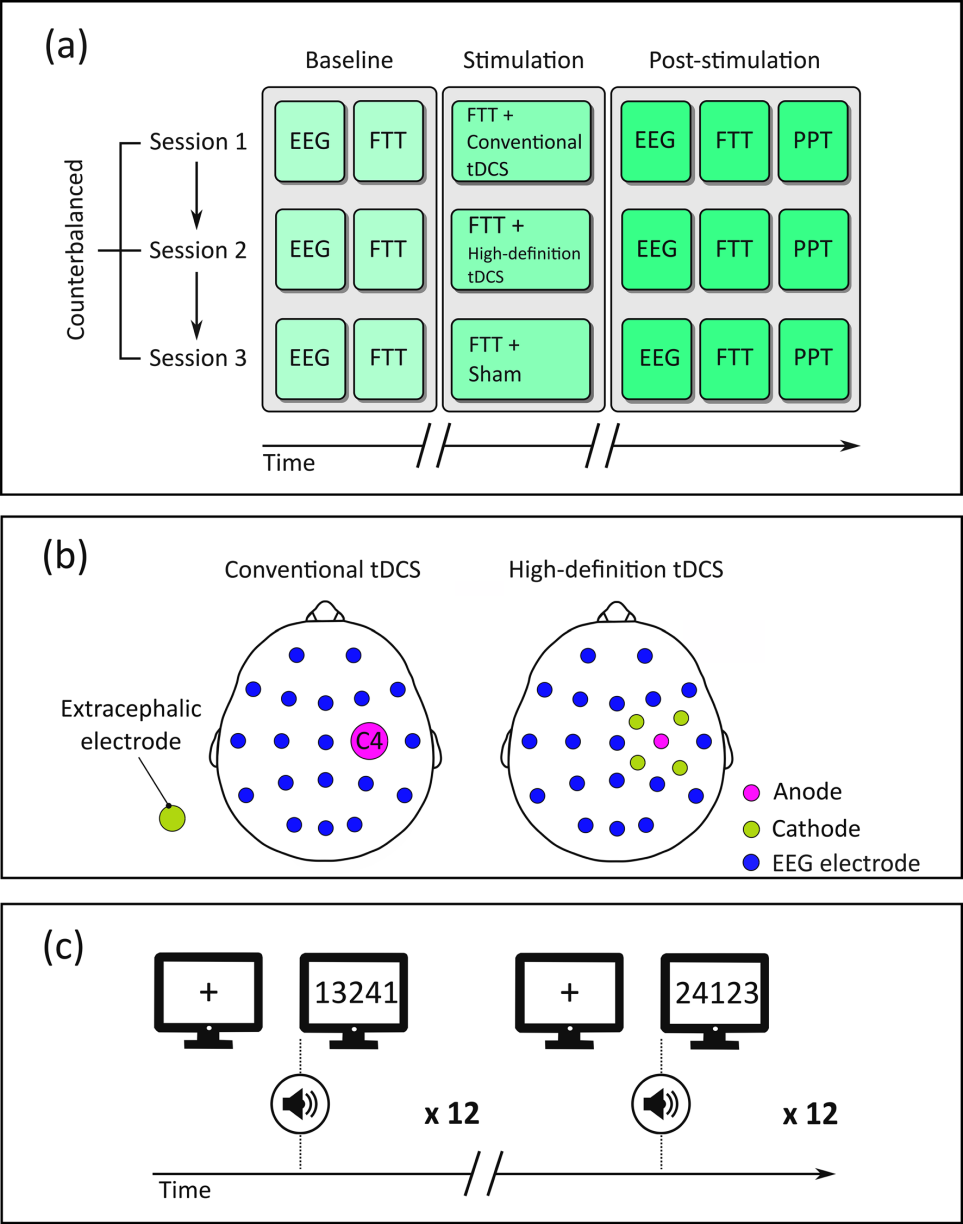
Experimental procedure, tDCS montages, and task. (**a**) Representation of the experimental procedure. Each participant took part to three experimental sessions that were separated by at least 6 days one another and where participants received Conventional tDCS, HD-tDCS, and sham. Blocks of the Finger Tapping Task were administered at the baseline, during the stimulation stage and at 5 min post-stimulation. Resting-state EEG was recorded at the baseline as well as after the stimulation stage. At the end of the experimental session the Purdue Pegboard Test was administered. (**b**) Head diagrams of Conventional and HD-tDCS electrode montages embedded in the EEG cap, which contained a total of 20 recording electrodes. In Conventional tDCS, the anode was placed on C4 and the cathode on the left shoulder. In HD-tDCS, the anode was placed on C4 and the cathodes on FC2, FC6, CP2, CP6. (**c**) Illustration of the Finger Tapping Task. Each trial started with the onset of a fixation point for 500 ms. Then, a sound was simultaneously presented with the string. Participants were asked to digit the same string 12 times in a row, after which a new string was presented. *FTT* Finger Tapping Task, *PPT* Purdue Pegboard Test.

The EEG signal was recorded with 20 Ag/AgCl sintered electrodes (StarStim, Barcellona) mounted on an elastic cap according to the International 10–20 system. The EEG electrodes were placed on the following sites: Fp1, Fp2, F7, F3, Fz, F4, F8, C3, Cz, C4, T7, T8, P7, P3, P4, P8, O1, O2 (Fig. 3). All recordings were referenced to the Pz electrode, while the ground electrode was placed on Oz. Raw data were digitalised with a frequency of 500 Hz.

### Tasks

The description of the behavioural tasks performed by participants (i.e., the Finger Tapping Task and the Purdue Pegboard Test) is available in supplementary material. Figure 3 shows a representation the Finger Tapping Task.

### Procedure

Participants were involved in three experimental sessions (Conventional tDCS, HD-tDCS, and sham), carried out on separate days and separated by a washout period lasting between 6 and 16 days. Importantly, the stimulation conditions were counterbalanced within the three experimental sessions. Each experimental session was divided into six steps.

Firstly, the EEG headcap was placed on the scalp. All impedances were kept below 5 kΩ. The session started with 5 min of resting-state EEG. During the EEG recording, participants were asked to stare a fixation point kept at 60 cm distance. Successively, participants performed the Finger Tapping Task (baseline stage) without any EEG recording. After this stage, they were invited to perform the Finger Tapping Task for 20 min (stimulation stage) while being delivered the stimulation (Conventional tDCS, HD-tDCS, or sham). The stimulation condition lasted for 20 min, meaning that the real stimulation (i.e., Conventional tDCS or HD-tDCS) or sham was delivered for the entire duration of the Finger Tapping Task. Regardless of the stimulation condition, the current strength was 1.5 mA with a ramp up/ramp down time of 30 s. In the sham condition, the current linearly increased for the first 30 s up to a 1.5 mA and then decreased to 0 mA in the next 30 s. After the stimulation stage, 5 min of resting-state EEG were recorded, following the aforementioned procedure. At the end of the EEG recording, participants performed the Finger Tapping Task for a third time (post-stimulation stage). Finally, the Purdue Pegboard Test was administered. Figure 3 shows a representation of the procedure.

### Statistical analysis

All data were analysed using RStudio software^67^ (version 1.2) and packages lme4^68^, lmerTest^69^, car^70^, and emmeans^71^. Linear mixed effect models (LMMs) and generalised linear mixed effect models (GLMMs) were used. Significance of the fixed effects terms were assessed by means of *F*-test using Satterthwaite approximation^72^. Post-hoc pairwise contrasts were corrected with Tukey's multiple comparison test. For significant interactions between a continuous variable and a factor, estimated marginal means contrasts were performed at the level of the 1st, 2nd, and 3rd quartile of the continuous variable. All relevant data and R scripts are available at https://osf.io/j4acs/, while all models performed in the analysis are available in supplementary material.

In the covariate-adjusted models, post-stimulation measurements, both behavioural and neurophysiological, were adjusted for baseline levels^25, 73^.

### The Finger Tapping Task

Participant's performance at the Finger Tapping Task was evaluated in terms of RTs and accuracy. RTs were measured as the time interval between string onset and the typing of the fifth digit (Fig. 3). RTs below 100 ms were removed from the analyses, as well as the strings not correctly typed.

Accuracy was computed as the ratio between the number of correct strings and the total number of strings. Error and correct strings were dichotomously coded, respectively as 0 and 1. As a consequence, accuracy was analysed by using GLMMs for binomial data, using a logit link function^74^.

The models were fit to investigate the effects of tDCS on post-stimulation performance, considering baseline alpha or beta power as covariates. In the models, *stimulation condition* (Conventional tDCS, HD-tDCS, sham) and the covariates *alpha/beta power* at the *baseline stage* (see EEG analysis) were considered as fixed-effect factors, and *participant*, *stimulation condition,* and *string repetition* as random intercepts (Table S1, supplementary material).

In addition, participant's performance at the Finger Tapping Task was also evaluated with two additional models (supplementary material). In these models, a conventional approach to analysis was adopted, without adjusting them for baseline levels. In these models, response times and accuracy at the Finger Tapping Task were evaluated, by including in the models the factors *task stage* (baseline, stimulation, and post-stimulation stage) and *stimulation condition* (Convention tDCS, HD-tDCS, and sham).

### The Purdue Pegboard Test

Participants’ performance at the Purdue Pegboard Test was scored as the mean number of pins, collars, and washers placed in the board, accordingly to the task instructions.

Since the anode was placed over C4 (right hemisphere), we restricted the analysis only to the mean score at the subtest performed with the left hand. Differences among the stimulation conditions (Conventional tDCS, HD-tDCS, sham) were investigated by fitting LMMs. *Stimulation condition* (Conventional tDCS, HD-tDCS, sham) and the covariates *alpha/beta power* at the *baseline stage* were considered as the fixed effect factors, and *participant* was included as the random-effect (Table S1, supplementary material).

### EEG analysis

The EEG data were pre-processed offline with Brainstorm^75^ for Matlab R2017b (The Mathworks Natic, MA, USA). First, continuous EEG was band-pass filtered with a cut-off frequency of 0.1–47 Hz. Then, the continuous EEG signal was visually inspected and channels with noise signal were removed. Independent component analysis^76^ was performed to correct the remaining artifacts (muscle activity and eye blinks). All independent components were visually inspected in terms of scalp distribution, frequency, timing and amplitude^77^. The mean number of removed independent components was 1.93 (*SD* = 0.87). Afterwards, the EEG was segmented into 150 non-overlapping epochs of 2000 ms.

Baseline correction was performed by subtracting the mean voltage of the whole epoch. The EEG signal was re-referenced to the mean of all channels. Epochs containing data points exceeding the amplitude of − 100 mV/+ 100 mV were excluded from the analysis. An average of 8.47% epochs were excluded. Successively, power spectrum density (Welch’s method) was conducted to extract power [signal units/sqrt(Hz) × 10^–5^] relative to the two bands of interest: alpha band (8–12 Hz) and beta band (18–22 Hz). Data were averaged within each band and were log-transformed to reduce skewness. Only power extracted from the electrodes close to the stimulation site (i.e., F4, Cz, T8, P4), and from C4 was considered in the analysis.

To investigate the effects of the stimulation conditions (Conventional tDCS, HD-tDCS, sham) on alpha/beta power at the post-stimulation stage, two LMMs were conducted. *Stimulation condition* (Conventional tDCS, HD-tDCS, sham) and the covariate *alpha/beta power* at the *baseline stage* were considered as fixed-effect factors. Random structure of the models consisted of *participant* and s*timulation condition* (Table S1, supplementary material).

## Supplementary Information

Supplementary Information.

## Data Availability

All relevant data and R scripts are available at https://osf.io/j4acs/, while the description of tasks (i.e., the Finger Tapping Task and the Purdue Pegboard Test) as well as all the models performed in the analysis are available in supplementary material.
