# Supplementary material for: Neurophysiological and behavioural effects of conventional and high definition tDCS
Source: Sci Rep. 2021 Apr 7;11:7659. doi: 10.1038/s41598-021-87371-z (PMC8027218; doi:10.1038/s41598-021-87371-z)
Supplement: Supplementary file 1 — Supplementary Information. [file 41598_2021_87371_MOESM1_ESM.pdf]

Supplementary material

Neurophysiological and behavioural effects of Conventional and High definition tDCS

Fabio Masina[a,b,1*], Giorgio Arcara[a,1], Eleonora Galletti[c], Isabella Cinque[c], Luciano Gamberini[b,c], Daniela Mapelli[b,c]

[a] IRCCS San Camillo Hospital, Venice, Italy
[b] Human Inspired Technologies Research Center, University of Padova, Italy
[c] Department of General Psychology, University of Padova, Italy

[1] Both authors contributed equally to this work

[*]Corresponding author. IRCCS San Camillo Hospital, Venice, Italy.
E-mail address: fabio.masina@ospedalesancamillo.net

Summary

The present supplementary material has intended to provide additional information to the manuscript. This material is divided into a series of parts:

1. **Statistical analysis** - This part reports all models conducted in the study.
2. **Finite element method (FEM) simulations** - This part shows a comparison of several electric field models simulated with SimNIBS.
3. **Tasks description** - This part provides a comprehensive description of tasks administered in the study.

Statistical analysis

Linear mixed effect models (LMMs) and generalized linear mixed effect models (GLMMs) were used to evaluate tDCS-induced effects. Significance of the fixed effects terms were assessed by means of *F*-test using Satterthwaite approximation. Post-hoc pairwise contrasts were corrected with Tukey's multiple comparison test. In case of a significant interaction between a continuous variable and a factor, estimated marginal means contrasts were performed at the level of the 1st, 2nd, and 3rd quartile of the continuous variable. Regarding the covariate-adjusted models in which the dependent variable was a behavioural measure (i.e., response times, accuracy, and Pegboard performance), we did not include, in the same model, power at the baseline and at the post-stimulation stage due to the high collinearity of these two covariates (VIF > 10).
Table S1 summarizes all the covariate-adjusted mixed-effects models run in the study. Details of each model will be shown in the following paragraphs.

Table S1. The table summarizes the factors entered in the models, specifying the dependent variable and the fixed/random-effects. Noteworthy, the symbols (~, *, +, |) are the same used in the computing environment R.

Model	Dependent variable	Fixed-effects		Random-effects
		Covariate	Manipulated variable	
LMM	FTT - RTs (ms) post-stimulation ~	α power baseline	* Stimulation condition	+ Stimulation condition|Participant<br>+ String repetition
LMM	FTT - RTs (ms) post-stimulation ~	β power baseline	* Stimulation condition	+ Stimulation condition|Participant<br>+ String repetition
GLMM	FTT - Accuracy (%) post-stimulation ~	α power baseline	* Stimulation condition	+ Stimulation condition|Participant<br>+ String repetition
GLMM	FTT - Accuracy (%) post-stimulation ~	β power baseline	* Stimulation condition	+ Stimulation condition|Participant<br>+ String repetition
LMM	PPT - Left hand ~	α power baseline	* Stimulation condition	+ Participant
LMM	PPT - Left hand ~	β power baseline	* Stimulation condition	+ Participant
LMM	α power post-stimulation ~	α power baseline	* Electrode<br>* Stimulation condition	+ Stimulation condition|Participant
LMM	β power post-stimulation ~	β power baseline	* Electrode<br>* Stimulation condition	+ Stimulation condition|Participant

Abbreviations: Linear mixed effect model (LMM); Generalized linear mixed effect model (GLMM); Response times (RTs); Finger Tapping Task (FTT); Purdue Pegboard Test (PPT).

The Finger Tapping Task_Response times

Formula: RTs_post-stimulation ~ **α_power_baseline** * Stimulation_condition + (1 + Stimulation_condition | Participant) + (1 | string_repetition)

Type III Analysis of Variance Table with Satterthwaite's method

Effects	NumDF	DenDF	F value	Pr (>F)
α_power_baseline	1	67.765	1.3275	.2533
Stimulation_condition	2	29.84	2.9834	.06589
α_power_ baseline * Stimulation_condition	2	29.84	3.0526	.06221

Formula: RTs_post-stimulation ~ **β_power_baseline** * Stimulation_condition + (1 + Stimulation_condition | Participant) + (1 | string_repetition)

Type III Analysis of Variance Table with Satterthwaite's method

Effects	NumDF	DenDF	F value	Pr (>F)
β_power_baseline	1	67.659	.0843	.77239
Stimulation_condition	2	29.93	3.5122	.04264
β_power_ baseline * Stimulation_condition	2	29.93	3.5836	.04025

The Finger Tapping Task_Accuracy

Formula: ACC_post-stimulation ~ **α_power_baseline** * Stimulation_condition + (1 + Stimulation_condition | Participant) + (1 | string_repetition), family = binomial

Analysis of Deviance Table (Type III Wald chisquare tests)

Effects	DF	Chisquare	Pr (>Chisq)
α_power_baseline	1	.104	.7471
Stimulation_condition	2	.0799	.9609
α_power_ baseline * Stimulation_condition	2	.1076	.9476

Formula: ACC_post-stimulation **~ β_power_baseline** * Stimulation_condition + (1 + Stimulation_condition | Participant) + (1 | string_repetition), family = binomial

Analysis of Deviance Table (Type III Wald chisquare tests)

Effects	DF	Chisquare	Pr (>Chisq)
β_power_baseline	1	.0087	.9255
Stimulation_condition	2	.2989	.8612
β_power_ baseline * Stimulation_condition	2	.275	.8716

The Purdue Pegboard Test

Formula: PPT_LeftHand ~ **α_power_baseline** * Stimulation_condition + (1 | Participant)

Type III Analysis of Variance Table with Satterthwaite's method

Effects	NumDF	DenDF	F value	Pr (>F)
α_power_baseline	1	61.443	4.7972	.03231
Stimulation_condition	2	56.319	.1034	.90191
α_power_ baseline * Stimulation_condition	2	56.326	.1307	.87773

Formula: PPT_LeftHand **~ β_power_baseline** * Stimulation_condition + (1 | Participant)

Type III Analysis of Variance Table with Satterthwaite's method

Effects	NumDF	DenDF	F value	Pr (>F)
β_power_baseline	1	67.438	3.8598	.05358
Stimulation_condition	2	57.111	.4024	.67058
β_power_ baseline * Stimulation_condition	2	57.107	.375	.68894

EEG analysis

Formula: α_power_post-stimulation **~ α_power_baseline** * Stimulation_condition * Electrode + (1 + Stimulation_condition | Participant)

Type III Analysis of Variance Table with Satterthwaite's method

Effects	NumDF	DenDF	F value	Pr (>F)
α_power_baseline	1	218.1	300.2299	< .001
Stimulation_condition	2	52.89	9.6936	< .001
Electrode	4	333.15	.3522	.8425
α_power_ baseline * Stimulation_condition	2	52.88	9.8679	< .001
α_power_ baseline * Electrode	4	332.39	.628	.6428
Stimulation_condition * Electrode	8	332.36	.6462	.7386
α_power_ baseline * Stimulation_condition * Electrode	8	331.96	.6911	.6994

Formula: β_power_post-stimulation **~ β_power_baseline** * Stimulation_condition * Electrode + (1 + Stimulation_condition | Participant)

Type III Analysis of Variance Table with Satterthwaite's method

Effects	NumDF	DenDF	F value	Pr (>F)
β_power_baseline	1	295.75	279.4053	< .001
Stimulation_condition	2	80.54	15.6444	< .001
Electrode	4	338.55	.6754	.6094
β_power_ baseline * Stimulation_condition	2	80.68	15.4481	< .001
β_power_ baseline * Electrode	4	338.45	.7417	.5641
Stimulation_condition * Electrode	8	339.95	.097	.9993
β_power_ baseline * Stimulation_condition * Electrode	8	339.94	.1092	.9989

Additional models

In addition to the above-mentioned models, participant's performance at the Finger Tapping Task was also evaluated with two additional models. In these models, we adopted a conventional approach to analysis, without adjusting the models for baseline levels. These models evaluated response times and accuracy at the Finger Tapping Task, by including in the models the factors *task stage* (baseline, stimulation, and post-stimulation stage) and *stimulation condition* (Convention tDCS, HD-tDCS, and sham).

Additional models_The Finger Tapping Task_Response times

Formula: RTs ~ Task_stage * Stimulation_condition + (1 + Task_stage | Participant) + (1 + Stimulation_condition | Participant) + (1 | string_repetition)

Type III Analysis of Variance Table with Satterthwaite's method				
Effects	NumDF	DenDF	F value	Pr (>F)
Task_stage	2	29	110.8594	< .001
Stimulation_condition	2	29.4	0.2122	.810004
Task_stage * Stimulation_condition	4	29911.6	3.8545	.003914

Additional models_The Finger Tapping Task_Accuracy

Formula: ACC ~ Task_stage * Stimulation_condition + (1 + Task_stage | Participant) + (1 + Stimulation_condition | Participant) + (1 | string_repetition), family = binomial

Analysis of Deviance Table (Type III Wald chisquare tests)			
Effects	DF	Chisquare	Pr (>Chisq)
Task_stage	2	2.4695	.290906
Stimulation_condition	2	.0944	.953877
Task_stage * Stimulation_condition	2	14.3584	.006235

With regard to RTs, a main effect of *task stage* was found [$F(2,29) = 110.86$, $p < .001$]. Post-hoc contrasts showed significant differences between the baseline and stimulation stage (1939 ms vs. 1709 ms, $p < .001$), the baseline and post-stimulation stage (1939 ms vs. 1548 ms, $p < .001$), and the stimulation and post-stimulation stage (1709 ms vs. 1548 ms, $p < .001$). In addition, a *task stage* * *stimulation condition* interaction was found [$F(4,29911.6) = 3.85$, $p = .0039$]. Post-hoc tests confirmed the differences among the baseline, stimulation, and post-stimulation stage, regardless of the stimulation condition (all $p < .001$), (Figure S1).

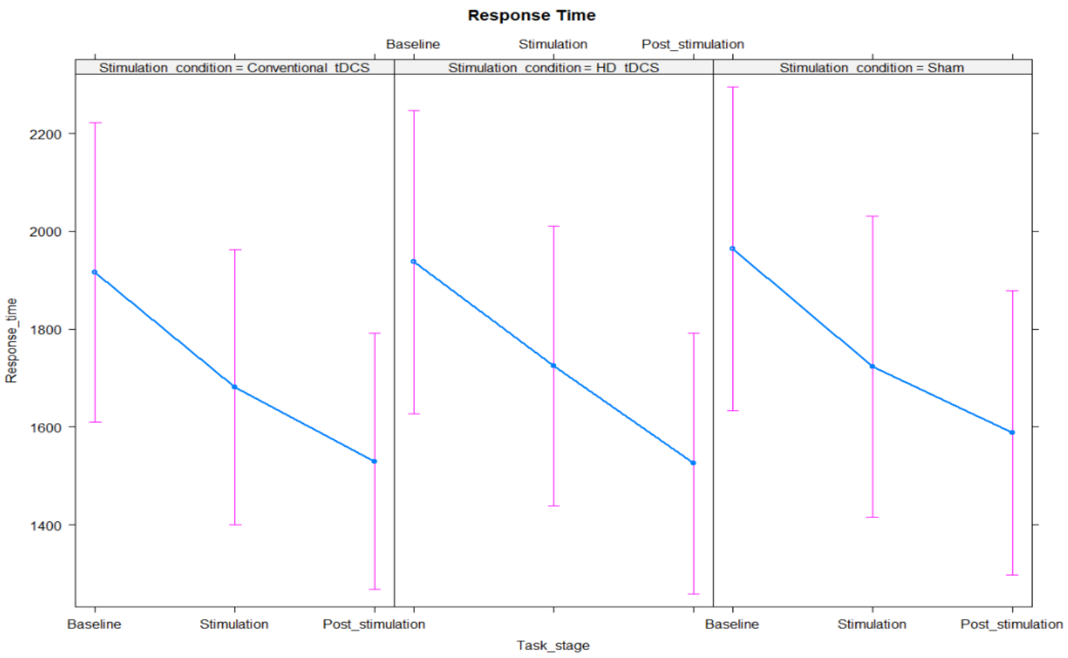

Figure S1. Results from the supplementary model evaluating response times. The interaction between *task stage* and *stimulation condition* showed a general reduction of response times across the task stages independently from the stimulation condition.

Regarding accuracy, a *task stage* * *stimulation condition* interaction was found [Wald $\chi^2(4) = 14.36$, $p = .006$]. Post-hoc tests showed a difference between the stimulation and post-stimulation stage in HD-tDCS condition ($p = .0054$, Figure S2). In addition, a difference between HD-tDCS and sham was found, only in the post-stimulation stage ($p = .0462$, Figure S3).

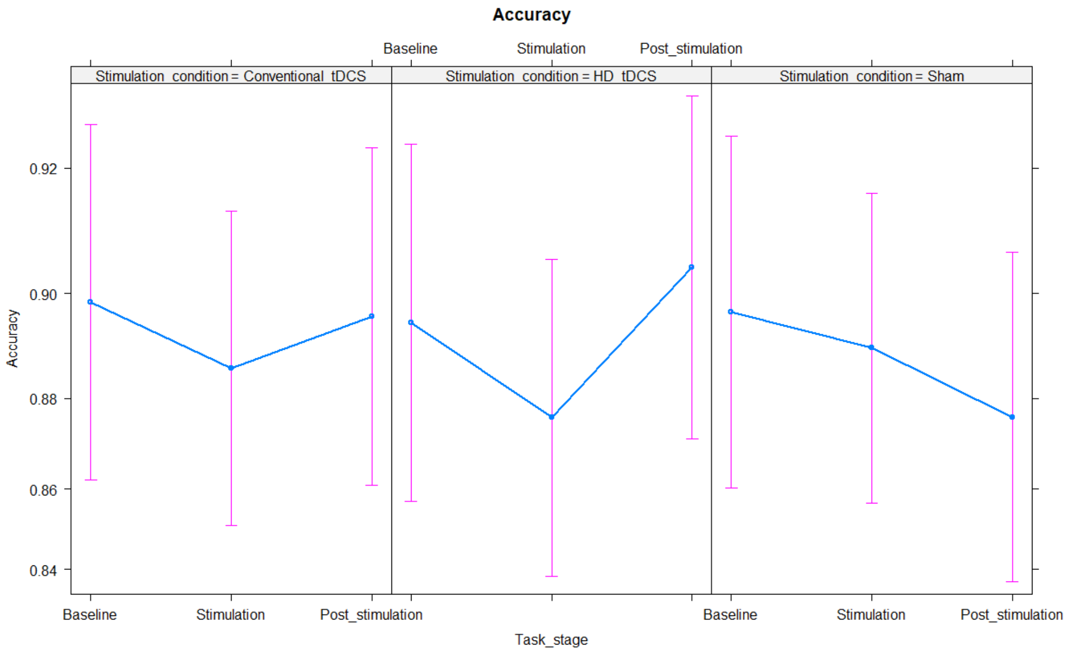

Figure S2. Results from the supplementary model evaluating accuracy. The interaction between *task stage* and *stimulation condition* showed an improvement of accuracy in the post-stimulation stage compared to the stimulation stage, only in HD-tDCS condition.

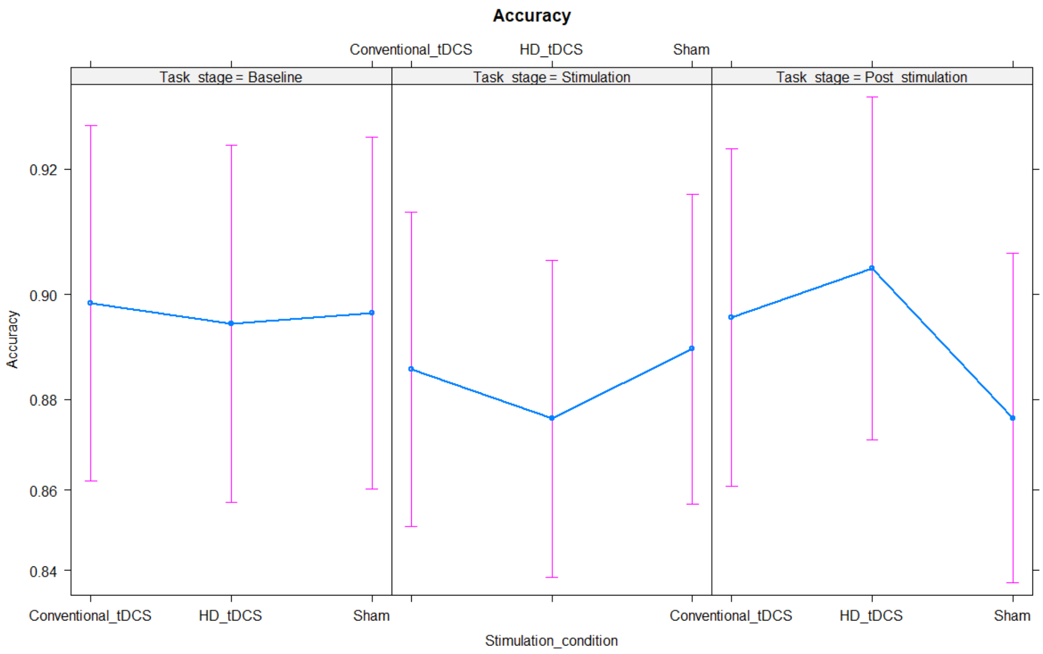

Figure S3. Results from the supplementary model evaluating accuracy. The interaction between *task stage* and *stimulation condition* showed a reduction of accuracy in sham compated to HD-tDCS, only in the post-stimulation stage.

The first model evaluating response times showed a general reduction of response times across the task stages, revealing a practise effect of participants. As for the second model considering accuracy at the Finger Tapping Task, it revealed an improvement of performance in the post-stimulation stage compared to the stimulation stage, only in the HD-tDCS condition. In addition, this model showed a reduction of accuracy in sham compated to HD-tDCS, only in the post-stimulation stage.

We report these supplementary models for completeness. However, we recommend considering results from these supplementary models with caution, especially the interaction between task stage and stimulation condition in the model evaluating accuracy. In fact, as argued in the manuscript, their effects may be biased since they assume statistical assumptions not always true (e.g., baseline imbalance).

Finite element method (FEM) simulations

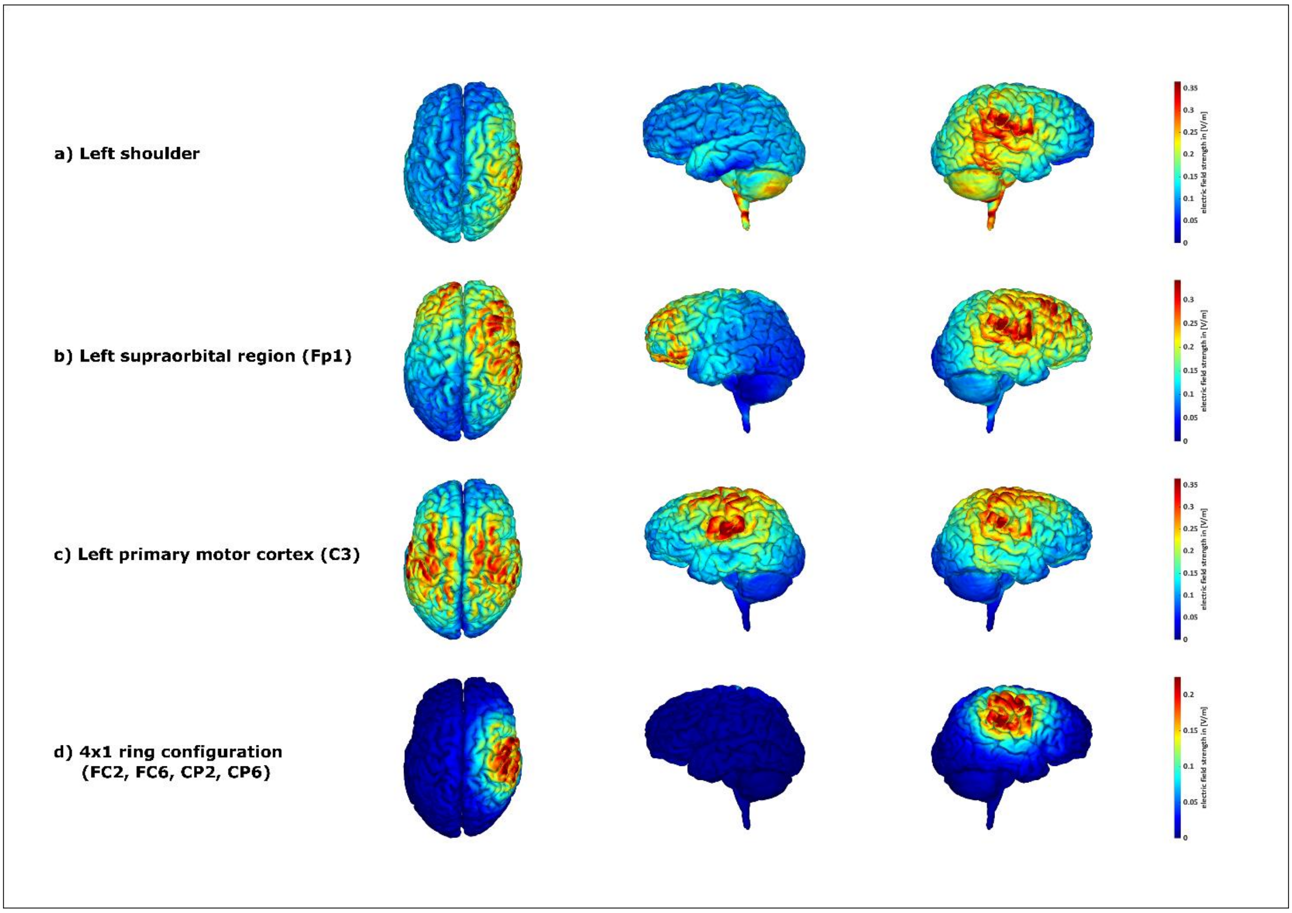

Figure S4. Finite element method (FEM) simulations showing the electric fields of three Conventional montages (i.e., a, b, c) and one HD-tDCS montage (i.e., d), all estimated by SimNIBS. On the left, the position of the return electrode/s. The figure shows the following simulations: a) Conventional tDCS (anode: C4; cathode: left shoulder); b) Conventional tDCS (anode: C4; cathode: left supraorbital region); c) Conventional tDCS (anode: C4; cathode: left primary motor cortex); d) HD-tDCS (anode: C4; cathodes: FC2, FC6, CP2, CP6). The simulations “a” and “d” depict the electric fields of the montages implemented in the study. As shown, the electric field elicited by HD-tDCS is much more focal than Conventional tDCS montages where the current is more spread. However, placing the return electrode of Conventional tDCS outside the brain (i.e., a) induces a unilateral polarization of the right hemisphere, similarly to HD-tDCS montage (i.e., d).

Tasks description

The Finger Tapping Task

Participants' dexterity was measured with a modified version of the Finger Tapping Task. After a central fixation point lasting 500 ms, a 5-digit numerical string appeared at the centre of a computer screen simultaneously with a brief sound warning participants to start typing as fast and correctly as they could, using their left hand. Each string could contain the numbers "1", "2", "3" or "4". No strings ever had two consecutive identical numbers (e.g. 3 2 2 1 4). The same string was presented 12 times in a row, after which a different string followed. The strings were randomly generated offline with Matlab R2017b (The Mathworks Natic, MA, USA) and then presented with E-Prime software (Psychological Software Tools, Pittsburgh, PA, USA; version 2.0.8.90).
Participants were required to type the strings respecting a number-finger rule. Specifically, they were asked to press the key "1" with the pinkie, "2" with the ring finger, "3" with the middle finger, and "4" with the index finger.
During string typing, the string was kept on the screen until participants finished typing it. Moreover, a little black dot appeared below the respective digit, to track how many numbers they had already typed.
In each session, participants performed the task in three stages: baseline, stimulation, and post-stimulation. In the baseline and post-stimulation stages (5 minutes), the task consisted of 5 different strings, each of them repeated 12 times in a row (60 trials), while in the stimulation stage (20 minutes), it consisted of 22 different strings repeated 12 times in a row (264 trials). The stimulation stage was divided into two blocks (132 trials each one). Since participants performed the task 9 times (3 stages x 3 sessions), parallel versions of the task with different strings were created and administered.

The Purdue Pegboard Test

The Purdue Pegboard Test was used to assess dexterity in a non-computer based fashion. The test consists of a board with two parallel rows with 25 little holes each. In the upper part of the board, there are 4 cups containing different pieces for the assembly (i.e. pins, collars, washers). The test is divided into 4 subtests during which participants have to perform several tasks (e.g. pick up one pin at a time with their left hand from the left-handed cup and place each pin in the left-handed row). The overall duration of the test was about 10 minutes.
